# Parent and Peer Influence on Recreational Use of Pain Medication: Are Their Influences Similar to That of Marijuana Use?

**DOI:** 10.1155/2013/236249

**Published:** 2013-08-28

**Authors:** Sasha Fleary, Aaron Taylor, Robert W. Heffer, E. Lisako McKyer

**Affiliations:** ^1^Eliot-Pearson Department of Child Development, Tufts University, 105 College Avenue, Medford, MA 02155, USA; ^2^Department of Health and Kinesiology, Dulie Bell, College Station, TX 77843, USA

## Abstract

Parent and peer disapproval were examined as potential predictors of recreational use of over-the-counter (OTC) and prescription pain medication. Risk perception was studied as a potential mediator of the effects of parent and peer disapproval. Four hundred and sixty-five college students (*M*_age_ = 18.57, SD = 0.86) were recruited between September 2009 and September 2010. Participants completed an online survey about their recreational medication use, other substance use, and correlates of use. Path analyses showed that predictors of OTC and prescription pain medication recreational use are largely similar to predictors of marijuana use in college students such that risk perception mediated both the effect of parent and peer disapproval on dichotomous misuse, and peer disapproval had a significant direct effect on dichotomous misuse. Prevention interventions for recreational use of pain medication should target risk perception and peer disapproval.

## 1. Introduction

Over-the-counter (OTC) and prescription drug misuse are the nonmedical use of medication, which is used without a prescription or for the feeling caused by the drug [1]. Motivations for misuse include alertness and concentration effects and getting high [2–4]. OTC and prescription drugs are fast becoming the drugs of choice for adolescents, with the National Center on Addiction and Substance Abuse [5] reporting a 212% increase in prescription drug misuse among adolescents between the 1992 and 2003 period. The National Survey on Drug Use and Health [6] and the Drug Abuse Warning Network [7] have also documented an increase in OTC drug misuse since 1992. Prescription drug misuse has increased independent of and relative to illicit drug use. Compton and Volkow [8] have reported that pain medication is second only to marijuana in being the most popular drug of choice for adolescents. Given that parent and peer influence have been highlighted as major predictors of substance use in adolescents [9] and that pain medications are the most widely abused prescription drugs [10], we hypothesize that parent and peer influence will affect pain medication misuse, specifically misuse to get high (recreational use), in the same way these influences affect marijuana use. 

Very little is known about what is responsible for the increase in prevalence and incidence rates of prescription drug misuse. It has been suggested that adolescents' and young adults' OTC and prescription drug misuse could be explained by their perception that such drugs are safer than illicit drugs, by the ease of access to drugs and by lower societal stigma [11–17]. Additionally, because OTC drugs are easily purchased, they can be used as an alternative when a prescription drug misuser is unable to access their preferred drug [18] as well as mixed with alcohol to achieve a “high” state [7]. Some researchers have shown that there is a strong relationship between illicit drug use and prescription drug misuse [13, 19–24]. Additionally, Ford [25] highlighted the importance of family and school bonds as protective factors against misuse. Ford [26] also suggested that adolescents whose parents and peers disapprove of substance use and whose peers use drugs are more likely to engage in prescription drug misuse.

Marijuana use, on the other hand, has been thoroughly studied, and clear correlates of marijuana use in adolescents have been identified across the literature. Specifically, sensation-seeking, risk perception of use, parent and peer attitudes towards use, adolescent and peers' delinquent behavior, peer use, adolescent tobacco and alcohol use, and demographic variables including gender and ethnicity [27–32] have all been shown to be related to adolescent marijuana use.

**Figure 1:**
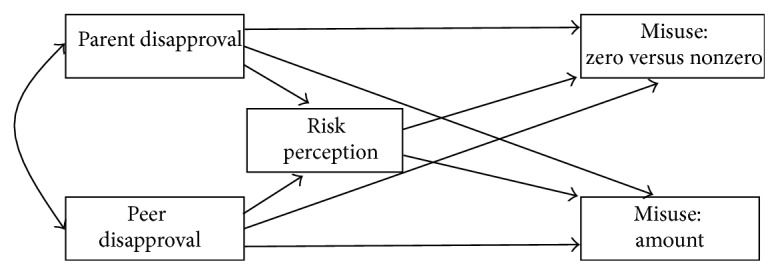
Path analysis model.This figure represents the paths to be tested for over-the-counter pain medication misuse, prescription pain medication misuse, and marijuana use. There are two models to be tested. One model identifies misuse: zero versus nonzero use (i.e., dichotomized use into users and nonusers) as the outcome variable and the other model identifies misuse: amount (reported use on frequency scale) as the outcome variable.

To better understand the relationship between illicit drug use and pain medication recreational use, we explored the extent to which three known predictors of marijuana use were similarly predictive of OTC and prescription pain medication recreational use. Specifically, we explored the extent to which risk perception, parent disapproval, and peer disapproval were predictive of recreational use and whether risk perception mediated the effects of parent and peer disapproval. Parent and peer disapproval have been strongly linked to risk perception and use in the marijuana literature [9, 28, 31, 33], hence their inclusion in the model (see Figure 1). Additionally, risk perception has been indicated in the health risk behaviors literature and alluded to in the prescription drug literature as a contributing variable in adolescents' decisions to engage in these behaviors [12, 14, 27, 34]. We examined the extent to which these relationships were gender dependent as the marijuana use literature has suggested that different variables are predictive of males' and females' use [28]. Additionally, because illicit drug use is correlated with OTC and prescription drugs misuse [13, 20, 21], we propose that the relationship between risk perception, parent and peer disapproval, and OTC and prescription pain medication recreational use will differ for marijuana users and nonusers.

This study differs from Ford, [26] in that our measures of parents' and peers' disapproval of misuse (specifically recreational use) are specific to OTC and prescription drug recreational use independent of other substance use. This is especially important since previous research has shown that perceptions of prescription drug misuse often differ from perceptions of illicit drug use [15, 17], and these differing perceptions will subsequently affect peers' and possibly parents' level of disapproval. Additionally, the inclusion of risk perception as a predictor variable provides some clinical utility to the study, in that it investigates the role of an easily targetable variable for intervention to prevent or decrease misuse. The inclusion of separate path analyses for OTC pain medication recreational use, prescription pain medication recreational use, and marijuana use also provides for a comparison of predictors of use of the different drugs. 

## 2. Methods

### 2.1. Participants

The sample consisted of 465 college students between the ages of 18 and 24 years (*M*_age_ = 18.57, SD = 0.86). The majority of participants (91%) were 18 or 19 years of age; the remaining 9% were ages 20–24 years. Participants were predominantly Caucasian (74%) and female (61%). Nineteen percent of participants (*n* = 88) reported some (nonzero) lifetime OTC or prescription drug recreational use with 13% (*n* = 60) and 11% (*n* = 50) reporting OTC and prescription pain medication recreational use, respectively. Twenty-nine percent of participants (*n* = 134) reported nonzero lifetime marijuana use.

### 2.2. Measures

Risk perception/perceived susceptibility was measured by perceived personal risk, which was the extent to which participants felt they would be at risk of getting sick or hurt if they recreationally used OTC pain medication, prescription pain medications, or marijuana, respectively. Risk perception was measured on a 7-point evaluation scale ranging from *no risk at all *(1) to *very much at risk *(7). To assess for parents' and peers' disapproval, participants were asked to report on how they felt their parents and peers would feel about them engaging in the recreational use of OTC pain medication, prescription pain medication, or marijuana on a 5-point Likert scale ranging from *strongly approve *(1) to *strongly disapprove *(5). Recreational use or misuse of OTC and prescription pain medication was defined as use of medications to get high. Marijuana misuse was defined as any marijuana use. Participants were asked about their lifetime misuse of OTC pain medication, prescription pain medication, and marijuana use on a 5-point frequency scale (see Table 1). Responses included *never* (1), 1–5 *times *(2), 6–19 *times *(3), 20–40 *times *(4), and *more than *40 *times *(5). OTC pain medication recreational use included the misuse of OTC Tylenol, Motrin, Advil, Aleve, Ibuprofen, and aspirin. Prescription pain medication recreational use included the misuse of Vicodin, Codeine, Oxycontin, and Percocet. Participants also reported on their recreational use of other classifications of OTC and prescription drugs including tranquilizers, stimulants, sedatives, steroids, and cough and cold syrup, although these items were not included in the analyses.

### 2.3. Procedures

 This study was approved by the authors' university Institutional Review Board. Data was collected as part of a larger project, “Media influences on health risk behaviors and prescription drug use in young adults.” Data was collected using a survey developed by the authors specifically for the project. College students were recruited from introductory psychology courses to participate in the study. Participants were invited to complete an online survey at computer workstations separated by desk partitions. The survey was administered via http://www.surveymonkey.com/. The online survey took approximately 35–50 minutes to complete, and participants received research credit for their class upon completion of the survey. To ensure participant privacy, respondent identification numbers were assigned.

### 2.4. Statistical Analyses

Descriptive statistics are shown in Table 2 (for OTC pain medication and prescription pain medication variables) and Table 3 (for marijuana variables). Path models were estimated separately for each set of variables for one of the three drugs: OTC pain medication, prescription pain medication, and marijuana. All path models took the same form, which is shown in Figure 1. Parent and peer disapproval of misuse of a drug were used to predict risk perception for that drug. Both disapproval variables and risk perception were then used to predict misuse of the drug. Misuse of the drug was entered using two variables, one representing whether a respondent reported zero misuse of the drug versus nonzero misuse and the other representing amount of use for respondents who reported a nonzero amount. This model allowed for the testing of whether risk perception mediated the effects of the disapproval variables on the misuse variables, which was done using the joint significance test [35].

For each drug, the path model was first estimated using data from all participants. It was then of interest to test whether any of the associations among the variables differed as a function of gender or as a function of use or nonuse of the other drugs. Multiple groups analyses were used to answer these questions. Each path model was reestimated first with gender as a grouping variable and second with misuse of marijuana (for OTC and prescription pain medication models) or OTC and prescription pain medication misuse (for the marijuana model). In order to be used as grouping variables, misuse variables were dichotomized into zero use versus nonzero use. Because OTC and prescription pain medication misuse were combined into a single grouping variable, a zero represented no misuse of either type and respondents reporting any use of either type, were scored as nonzero. Path analysis models were estimated using Mplus 6.0 [36]. The misuse-dependent variables were each entered using two variables in the model by way of the TWOPART feature of Mplus, which scores all participants on a dichotomous use variable depending on whether they reported zero or nonzero misuse and also score amount of misuse as a separate continuous variable. Amount of misuse is scored as missing for respondents who have zero use, but data from these participants was still able to be included in the models because Mplus can estimate models using full information maximum likelihood, which can include partially complete data. As the amount of misuse was skewed, the MLR estimator, a variation of maximum likelihood that is more robust to nonnormal data than the more typical maximum likelihood, was used to estimate the models.

## 3. Results

 Regarding descriptive statistics, parent disapproval was positively correlated with peer disapproval and risk perception for marijuana and OTC pain and prescription pain medication misuse variables. Parent disapproval for OTC pain medication was also negatively correlated with marijuana use and lifetime use. Among marijuana use variables, parent disapproval was negatively correlated with lifetime use; however, this was only true for participants who did not report any pain medication misuse. Peer disapproval was positively correlated with risk perception and negatively correlated with lifetime use for marijuana and OTC pain and prescription pain medication misuse variables. Peer disapproval of OTC pain and prescription pain medication misuse was also negatively correlated with marijuana use. Risk perception was positively correlated with gender and negatively correlated with lifetime use and marijuana use for both types of pain medication variables. For marijuana variables, risk perception was negatively correlated with lifetime use. However, risk perception was only positively correlated with gender among participants who reported no pain medication misuse. Lifetime use was positively correlated with marijuana use and negatively correlated with gender for the prescription pain variables.

Because Mplus uses numerical integration to estimate models with dichotomous-dependent variables, it does not report the usual fit statistics or fit indices for such models. Fit of the models was not an issue, though, as they included all possible associations among the variables (note that no association between the two misuse variables could be included because the continuous variable only took on nonmissing values for respondents scoring one on the dichotomous variable) and so were effectively saturated. Note that because the data is cross-sectional, the term “predictor” is used for statistical purposes rather than to indicate causality.

Results for the path models are shown in Table 4. For OTC pain medication misuse, peer disapproval (*β* = .516, *P* < .05) significantly predicted risk perception, with higher levels of peer disapproval predicting higher levels of risk perception. Both peer disapproval (*β* = −.401, *P* < .05) and risk perception (*β* = −.221, *P* < .05) significantly predicted dichotomous misuse, with higher levels of peer disapproval and risk perception predicting lower likelihood of nonzero misuse. Because peer disapproval predicted risk perception and risk perception predicted dichotomous misuse, risk perception was a significant mediator of the effect of peer disapproval on dichotomous misuse. There were no significant predictors of amount of misuse for respondents who reported a nonzero amount. Multiple groups analyses revealed no significant differences in path coefficients as a function of either gender groups (*χ* ^2^(8) = 3.44, *P* > .05) or groups defined by zero or nonzero marijuana misuse (*χ* ^2^(8) = 7.60, *P* > .05).

For prescription pain medication misuse, both parent disapproval (*β* = .183, *P* < .05) and peer disapproval (*β* = .425, *P* < .05), significantly predicted risk perception, with higher levels of parent and peer disapproval predicting higher levels of risk perception. Both peer disapproval (*β* = − .424, *P* < .05) and risk perception (*β* = −.188, *P* < .05) significantly predicted dichotomous misuse, with higher levels of peer disapproval and risk perception predicting lower likelihood of nonzero misuse. Because peer disapproval predicted risk perception and risk perception predicted dichotomous misuse, risk perception was a significant mediator of the effect of peer disapproval on dichotomous misuse. Parent disapproval (*β* = .286, *P* < .05) significantly predicted amount of misuse for respondents who reported a nonzero amount, but in an unexpected direction: higher levels of disapproval predicted greater amounts of misuse. Multiple groups analyses revealed no significant differences in path coefficients as a function of either gender groups (*χ* ^2^(8) = 12.35, *P* > .05) or groups defined by zero or nonzero marijuana misuse (*χ* ^2^(8) = 3.13, *P* > .05).

For marijuana misuse, both parent disapproval (*β* = .162, *P* < .05) and peer disapproval (*β* = .493, *P* < .05) significantly predicted risk perceptions, with higher levels of parent and peer disapproval predicting higher levels of risk perception. Peer disapproval (*β* = − .356, *P* < .05) and risk perception (*β* = −.409, *P* < .05) significantly predicted dichotomous misuse, with higher levels of peer disapproval and risk perception predicting lower likelihood of nonzero misuse. Parent disapproval also significantly predicted dichotomous misuse, but this effect varied depending on whether respondents were in the zero misuse or nonzero misuse group for OTC and prescription pain medications, so it is discussed below. Both peer disapproval (*β* = −.262, *P* < .05) and risk perception (*β* = −.454, *P* < .05) were significant predictors of amount of misuse for respondents who reported a nonzero amount, with higher levels of disapproval and risk perception predicting smaller amounts of use. Because both parent and peer disapproval predicted risk perception and risk perception predicted both dichotomous misuse and amount of misuse, risk perception was a significant mediator of the effect of both parent and peer disapproval on both dichotomous misuse and amount of misuse. Multiple group analyses revealed no significant differences in path coefficients as a function of gender groups (*χ* ^2^(8) = 10.02, *P* > .05). There was a significant difference in path coefficients as a function of groups defined by zero or nonzero use of OTC or prescription pain medications (*χ* ^2^(8) = 18.81, *P* < .05). Tests of invariance of the individual paths revealed that the only path to differ significantly was for parent disapproval predicting dichotomous misuse. For respondents reporting zero OTC or prescription pain medication misuse, this association was significant and negative (*β* = −.131, *P* < .05), so higher levels of disapproval predicted lower likelihoods of nonzero misuse. For respondents reporting nonzero OTC or prescription pain misuse, this association was significant and positive (*β* = .282, *P* < .05), so higher levels of disapproval predicted higher likelihoods of nonzero misuse.

## 4. Comments/Discussion

To add to the understanding of OTC and prescription pain medication misuse and its relationship with other substance abuse in adolescents and young adults, we explored the relationship between parent and peer disapproval of recreational use, risk perception of recreational use, and reported recreational use. The results of this study indicate that similar to marijuana use, risk perception of OTC and prescription pain medication recreational use are influenced by parent and peer disapproval of recreational use, and use itself is influenced by peer disapproval and risk perception of recreational use.

Noteworthy was the ability of peer disapproval of misuse to predict dichotomous misuse over and above the mediated effect through risk perception, for all three drugs: OTC and prescription pain medication misuse and marijuana use. This is consistent with findings in the substance abuse literature [31, 33] and suggests that peer subculture may either encourage or act as a protective variable against adolescents engaging in the recreational use of all substances including prescription drugs. This also highlights the role of development in adolescents' decisions to engage in health risk behaviors; that is, they are in a period in their lives where peers are their key *counterplayers *and they may follow the norms outlined by their subgroup; hence, peer disapproval has more influence on their perceptions of harm and use [37]. These results also suggest that in order to increase risk perception and reduce recreational use of OTC and prescription pain medication, program planners should work towards reducing the acceptability of OTC and prescription pain medication misuse among adolescents, since they influence each others' perceptions. The Office of National Drug Policy [38] and Partnership for a Drug-Free America [15] launched a prescription drug abuse media campaign targeting parents and health and school professionals; however, no ads were directly marketed toward adolescents and young adults. Based on the significant influence of peer disapproval on risk perception and use, future campaigns should target adolescents and young adults.

Parent disapproval was predictive of risk perception of prescription pain medication recreational use and marijuana use. We speculate that adolescents may take their parents' opinions into consideration when formulating their risk perceptions on both marijuana and prescription pain medication as opposed to OTC pain medication because of the restrictive access to these substances; however, this hypothesis should be explored in future studies. Parent disapproval of misuse failed to directly predict dichotomous misuse of OTC and prescription pain medications (although it did have a mediated effect through risk perception for prescription pain medications). This highlights the need for parents and by extension other adults working with adolescents to focus less on preventing behavior via direct methods (e.g., “just say no” campaigns) and focus more on educating adolescents on the risks associated with the behavior. The goal of redirecting adults' focus on educating adolescents on the risks of OTC and prescription pain misuse is to influence other correlates of use, such as peer disapproval and risk perception, in order to prevent or decrease use.

Parent disapproval predicting dichotomous misuse of marijuana is consistent with the marijuana use literature [39, 40]. However, the finding of reverse relationships when the sample was divided into OTC and prescription pain drugs zero and nonzero misusers provides some additional data about the relationship of use for the top two drugs of choice for adolescents. Specifically, adolescents who engaged in OTC and prescription pain misuse were more likely to be marijuana users the more their parents disapproved of marijuana use, whereas those who were zero OTC and prescription pain misusers were less likely to engage in marijuana use if their parents disapproved of marijuana use. These results are suggestive of a deviant and high sensation-seeking subculture trend in adolescents who engage in both pain medication and marijuana use and this subculture is seen across the substance abuse literature [9, 31, 33]. It is possible that among this subculture, parent interventions may encourage adolescents to engage in substance use, providing further need for parents to focus less on preventing behavior and more on highlighting the risks associated with the behaviors. Another explanation may be that of reverse causality, whereby the more the adolescents engage in use, the more their parents disapprove of their behavior.

Although risk perception was significantly predictive of use in the general models, the regression weights and significance values suggest that it was a weaker predictor for OTC and prescription pain medication recreational use than for marijuana use. These findings suggest that although risk perception may play an important role in adolescents' decision making regarding substance use and other health risk behaviors [28, 34, 41, 42], risk perception is less of an important variable in adolescents' decision-making regarding recreational use of OTC and prescription pain medication. Additional variables that are uncommon to the substance abuse literature, such as lower societal stigma, and comparative risk perception to street drugs may be more important in adolescents' decision making regarding prescription drug recreational use [12, 14]. Additionally, risk perception may interact with other variables not studied here to explain adolescents' decision making regarding use (e.g., general attitudes toward prescription drug use, frequency of family and friends' medical use of prescription drugs). Future studies should explore how other correlates of recreational use of OTC and prescription pain medication interact with risk perception to influence misuse.

### 4.1. Limitations and Conclusions

This study was restricted to recreational users of OTC and prescription pain medication; these users do not encompass all nonmedical use; therefore, generalizations about misuse are limited. Future studies should test this model with a broader definition of nonmedical users. Another limitation regarding generalization is the use of a convenience sample of college students; future studies should test this model on a community sample. Another limitation of this study is that the psychosocial characteristics of the sample were not considered. Adolescents and young adults are highly influenced by developmental characteristics and their external environment and future studies should explore the extent to which developmental characteristics interact with these environmental variables to predict risk perception and use. Additionally, future studies should retest these paths with a larger sample (preferably including more participants who report nonzero use) to check for consistency of the results across samples. Future studies should also compare OTC and prescription pain medication misuse to other illicit drug use and other health risk behaviors. Finally, the present study was cross-sectional, so temporal precedence cannot be established for the assumed causal relationships between variables.

Despite these limitations, this study provides evidence that suggests that predictors of OTC and prescription pain medication recreational use are similar to predictors of marijuana use in adolescents in that peer disapproval is a significant predictor of risk perception and misuse. Additionally, both peer and parent disapproval are significant predictors of risk perception of prescription pain medication misuse. Unlike marijuana use, OTC and prescription pain medication misuse in adolescents are relatively less explained by risk perception suggesting that other variables may be responsible for adolescents' decision to engage in the misuse of OTC and prescription pain medication.

## Figures and Tables

**Table 1:** Frequency of useormisuse for OTC pain medication, prescription pain medication, and marijuana.

	OTC pain	Pain	Marijuana
Never	406 (86.9%)	417 (89.3%)	333 (71.3%)
1–5 times	41 (8.8%)	34 (7.3%)	69 (14.8%)
6–19 times	7 (1.5%)	10 (2.1%)	21 (4.5%)
20–40 times	4 (.9%)	3 (.6%)	11 (2.4%)
More than 40 times	9 (1.9%)	3 (.6%)	33 (7.1%)

OTC: over the counter.

**Table 2:** Descriptive statistics for OTC pain medication misuse and prescription pain medication misuse.

	OTC		(1) Parent	(2) Peer	(3) RP	(4) Life use	(5) Gender	(6) Marijuana	Prescription	
	M	SD							M	SD
(1) Parent disapproval	2.43	1.21		.359∗	.335∗	−.084	.010	.034	4.38	0.89
(2) Peer disapproval	3.19	1.33	.346∗		.491∗	−.353∗	.113∗	−.214∗	3.93	1.09
(3) Risk perception	3.61	2.00	.249∗	.540∗		−.266∗	.224∗	−.143∗	5.30	1.71
(4) Lifetime use	1.22	0.70	−.095∗	−.236∗	−.217∗		−.111∗	.318∗	1.16	0.54
(5) Gender	0.61	0.49	−.049	.011	.142∗	−.121∗		−.054	0.61	0.49
(6) Marijuana use^†^	0.29	0.45	−.102∗	−.174∗	−.098∗	.140∗	−.054		0.29	0.45

Correlations for OTC pain medication misuse variables are below the main diagonal. Correlations for prescription pain medication misuse variables are above the main diagonal. The asterisks indicate significant correlations.

OTC: over the counter; RP: risk perception.

^*^ *P* < .05.

^†^Marijuana use was dichotomized into zero use versus nonzero use because it was used as a grouping variable for multiple group analyses.

Grouping variable: a zero represented no misuse of either type, and respondents reporting any use of either type were scored as nonzero.

**Table 3:** Descriptive statistics for marijuana misuse by groups defined by any lifetime misuse of OTC or prescription pain medication versus no such misuse.

	No OTC/Prescription misuse (*n* = 377)		(1) Parent	(2) Peer	(3) RP	(4) Life use	(5) Gender	OTC/Prescription misuse (*n* = 88)	
	M	SD						M	SD
(1) Parent disapproval	4.86	0.44		.312∗	.305∗	−.047	.112	4.61	0.62
(2) Peer disapproval	3.67	1.32	.252∗		.626∗	−.574∗	.043	2.72	1.34
(3) Risk perception	5.36	1.74	.254∗	.465∗		−.604∗	.169	3.83	2.10
(4) Lifetime use	1.36	0.82	−.252∗	−.407∗	−.536∗		−.139	2.58	1.73
(5) Gender	0.63	0.48	.008	.078	.223∗	−.080		0.50	0.50

Descriptive statistics are shown separately for respondents who reported zero OTC or prescription misuse and respondents who reported nonzero OTC or prescription misuse because the path model for marijuana variables differed significantly depending on this grouping variable (see rightmost column). Correlations for respondents reporting zero OTC or prescription pain medication misuse are below the main diagonal. Correlations for respondents reporting nonzero OTC or prescription pain medication misuse are above the main diagonal. The asterisks indicate significant correlations.

OTC: over the counter; RP: risk perception.

^*^ *P* < .05.

**Table 4:** Standardized coefficients for path models for OTC pain medication, prescription pain medication, and marijuana.

Variables	Analysis		
	OTC	Prescription	Marijuana
DV: risk perception			
Parent disapproval	0.070	0.183∗	0.162∗
Peer disapproval	0.516∗	0.425∗	0.493∗
DV: lifetime misuse, zero versus nonzero			
Parent disapproval	0.032	0.011	−0.131∗/0.282^∗†^
Peer disapproval	−0.401∗	−0.424∗	−0.356∗
Risk perception	−0.221∗	−0.188∗	−0.409∗
DV: lifetime misuse, amount			
Parent disapproval	−0.040	0.286∗	0.037
Peer disapproval	0.286	−0.355	−0.262∗
Risk perception	−0.271	−0.100	−0.454∗

Each column has results for a separate path model. Lifetime misuse, zero versus nonzero, is a dichotomous variable, so the part of the model predicting this variable in each analysis is a logistic regression model.

DV: dependent variables; OTC: over the counter.

∗ *P* < .05.

^†^This path differed significantly for respondents depending on whether they reported zero OTC or prescription pain medication misuse (first value) versus nonzero use (second value).
